# Developmental ethanol exposure has minimal impact on cerebellar microglial dynamics, morphology, and interactions with Purkinje cells during adolescence

**DOI:** 10.3389/fnins.2023.1176581

**Published:** 2023-05-05

**Authors:** MaKenna Y. Cealie, James C. Douglas, Linh H. D. Le, Erik D. Vonkaenel, Matthew N. McCall, Paul D. Drew, Ania K. Majewska

**Affiliations:** ^1^Majewska Laboratory, Department of Neuroscience, School of Medicine and Dentistry, University of Rochester, Rochester, NY, United States; ^2^Drew Laboratory, Department of Neurobiology and Developmental Sciences, University of Arkansas for Medical Sciences, Little Rock, AR, United States; ^3^McCall Laboratory, Department of Biostatistics and Computational Biology, School of Medicine and Dentistry, University of Rochester, Rochester, NY, United States

**Keywords:** microglia, cerebellum, Purkinje cell, immune system, ethanol, fetal alcohol spectrum disorders (FASD), prenatal alcohol exposure, neurodevelopment

## Abstract

**Introduction:**

Fetal alcohol spectrum disorders (FASD) are the most common cause of non-heritable, preventable mental disability, occurring in almost 5% of births in the United States. FASD lead to physical, behavioral, and cognitive impairments, including deficits related to the cerebellum. There is no known cure for FASD and their mechanisms remain poorly understood. To better understand these mechanisms, we examined the cerebellum on a cellular level by studying microglia, the principal immune cells of the central nervous system, and Purkinje cells, the sole output of the cerebellum. Both cell types have been shown to be affected in models of FASD, with increased cell death, immune activation of microglia, and altered firing in Purkinje cells. While ethanol administered in adulthood can acutely depress the dynamics of the microglial process arbor, it is unknown how developmental ethanol exposure impacts microglia dynamics and their interactions with Purkinje cells in the long term.

**Methods:**

To address this question, we used a mouse model of human 3rd trimester exposure, whereby L7^cre^/Ai9^+/−^/Cx3cr1^G/+^ mice (with fluorescently labeled microglia and Purkinje cells) of both sexes were subcutaneously treated with a binge-level dose of ethanol (5.0 g/kg/day) or saline from postnatal days 4–9. Cranial windows were implanted in adolescent mice above the cerebellum to examine the long-term effects of developmental ethanol exposure on cerebellar microglia and Purkinje cell interactions using *in vivo* two-photon imaging.

**Results:**

We found that cerebellar microglia dynamics and morphology were not affected after developmental ethanol exposure. Microglia dynamics were also largely unaltered with respect to how they interact with Purkinje cells, although subtle changes in these interactions were observed in females in the molecular layer of the cerebellum.

**Discussion:**

This work suggests that there are limited *in vivo* long-term effects of ethanol exposure on microglia morphology, dynamics, and neuronal interactions, so other avenues of research may be important in elucidating the mechanisms of FASD.

## 1. Introduction

Fetal alcohol spectrum disorders (FASD) are the most common cause of non-heritable, preventable mental disability, occurring in almost 5% of births in the United States (May et al., 2014; Williams et al., 2015). There is no known cure for FASD, few therapies, and the mechanisms that underlie ethanol’s effects in development remain unclear. Ethanol exposure during gestation can cause developmental and central nervous system abnormalities, which result in a wide range of cognitive, behavioral, and physical impairments (Wilhelm and Guizzetti, 2015; Saito et al., 2016), such as facial dysmorphia, decreased brain size, and abnormal brain structure (Hamre and West, 1993; Napper and West, 1995; Idrus and Napper, 2012). The cerebellum appears to be particularly vulnerable, displaying ethanol-induced anatomical changes which could underlie deficits in behaviors, such as impaired motor coordination and learning, that are associated with FASD (Servais et al., 2007; Topper et al., 2015). These changes are long lasting, with smaller cerebellar volumes and motor skill impairments reported in adolescents and young adults exposed to ethanol during gestation (Safe et al., 2018; Sullivan et al., 2020). Ethanol may have a strong effect on cerebellar structure and function due to the cerebellum’s unique developmental timeline. Cerebellar development is protracted continuing into postnatal life, compared to areas like the cortex, where neurogenesis and migration are completed at birth in humans and mice (Stagni et al., 2015). The peak of cerebellar neurogenesis occurs postnatally in mice, around postnatal days (P) 4–9 (Stagni et al., 2015), which translates to a time of intense synaptogenesis in other brain regions, called the growth spurt period. This brain growth spurt period occurs in the third trimester in humans and may be a period of particular vulnerability to developmental ethanol exposure (Dobbing and Sands, 1979; Bayer et al., 1993; Wilhelm and Guizzetti, 2015; Saito et al., 2016).

One cerebellar cell type that develops during the early postnatal period in mice is the Purkinje cell, the sole output of the cerebellum. Around birth, Purkinje cells migrate and form the Purkinje Cell Layer (PCL) of the cerebellum (Altman and Bayer, 1978; Zhang and Goldman, 1996; Zhang et al., 2010). Initially the PCL is a multilayer but thins into a monolayer around P6 to P7 (Zhang et al., 2010). Postnatally, Purkinje cell dendrites grow and spread in the Molecular Layer (ML), with their distinct and elaborate dendritic fan shape growing by the third postnatal week (Berry and Bradley, 1976; Yamada et al., 2000). Other cerebellar cell types migrate, mature, and synapse with Purkinje cells throughout the first three postnatal weeks, making this a potential critical period for ethanol’s effects on the cerebellar circuit as a whole (Zhang et al., 2010; Rahimi-Balaei et al., 2018). Indeed, it has been reported that Purkinje cells are impacted by ethanol. Reduced numbers of Purkinje cells have been reported when rodents were given ethanol postnatally, particularly from P4–5 (Goodlett et al., 1990; Hamre and West, 1993; Goodlett and Eilers, 1997; Topper et al., 2015). Additionally, effects on the surviving Purkinje cells have been described, such as increases in simple spike firing rate, decreased intrinsic excitability, and decreased strength of excitatory parallel fiber-Purkinje cell synapses (Servais et al., 2007; Zamudio-Bulcock et al., 2014), suggesting a multifactorial effect of ethanol on the cerebellar circuit.

Microglia, the immune cells of the central nervous system, are also affected by developmental ethanol exposure. Ethanol can induce cell death of microglia and alterations in the phenotype of surviving microglia (Kane et al., 2011). Ethanol-exposed microglia undergo morphological changes, displaying a phenotype associated with immune activation, and release inflammatory factors that could contribute to cerebellar neuronal cell death and changes in firing patterns (Drew et al., 2015; Topper et al., 2015; Kane et al., 2021; Lowery et al., 2021), suggesting a role for microglia in ethanol-induced Purkinje cell defects. Microglia and neuroinflammation have been an area of increasing interest in the study of FASD (Kane et al., 2021; Komada and Nishimura, 2022). In the healthy brain, microglia have a surveillance role and play an integral part in the development of neurons and connectivity through the phagocytosis of apoptotic neurons and the pruning and formation of synapses (Dean et al., 2012; Nakayama et al., 2018; Kana et al., 2019; Perez-Pouchoulen et al., 2019; Yamamoto et al., 2019). Microglia make physical contacts with neuronal dendrites, axons, and somas, influencing synaptic plasticity and neuronal firing (Cserép et al., 2021). Microglia can also release factors that influence cellular and circuit assembly (Marin-Teva et al., 2004; Wong et al., 2017). Disruption of these functions, particularly in development, may have long-lasting consequences and contribute to FASD pathology.

Microglia are highly dynamic cells, whose motile arbor can shape neuronal circuit development and connectivity in the cerebellum (Stoessel and Majewska, 2021). Microglia display regional heterogeneity, with cerebellar microglia displaying phenotypes that make them distinct from many other microglial populations. For instance, on a transcriptomic level, cerebellar and hippocampal microglia exhibit a more immune alert state and express more genes related to energy metabolism than cortical microglia (Grabert et al., 2016). Structurally, cerebellar microglia are less densely distributed and appear less ramified than those in the visual cortex (Stowell et al., 2018). Dynamically, cerebellar microglia survey less territory but display a unique somal motility *in vivo* (Stowell et al., 2018). In adulthood, microglia in the ML and PCL can contact and wrap Purkinje cell dendrites and somas (Stowell et al., 2018). If similar dynamic interactions between microglia and Purkinje cells occur during development, they could potentially influence processes that shape the development of cerebellar neurons and connectivity. In adulthood, acute ethanol administration resulted in microglia that were hyper-ramified and displayed decreased motility and surveillance in the PCL, whereas no effects were seen in the ML, suggesting a layer-specificity in microglial vulnerability (Stowell and Majewska, 2020). However, it is unknown if developmental ethanol exposure can affect microglia dynamics and their interactions with Purkinje cells later in life.

Here, we examine whether ethanol exposure in development can affect cerebellar microglia dynamics, morphology, and neuronal interactions in adolescence. While there is evidence that both microglia and Purkinje cells are altered by early ethanol exposure, there is currently no information on whether the interactions between these two cells also change. We attempt to fill this gap and examine if and how these interactions are altered by developmental ethanol using *in vivo* two-photon imaging. We use a mouse model of FASD, in which mice are exposed to binge-level amounts of ethanol from P4 through P9, the time period equivalent to the human third trimester. We found that *in vivo* microglia dynamics, motility and surveillance, as well as microglia morphology were relatively unchanged in adolescence after developmental ethanol exposure. Subtle differences in dynamic cell–cell interactions between Purkinje cells and microglia may have been triggered by ethanol specifically in females in the molecular layer. This work suggests that high levels of ethanol exposure during the equivalent of the third trimester does not have profound effects on the structure and dynamics of cerebellar microglia or their interactions with Purkinje cells in early adolescence.

## 2. Materials and methods

### 2.1. Animals

All experimental protocols were carried out in strict accordance with the University of Rochester Committee on Animal Resources and National Institutes of Health Guidelines. All transgenic mice (*N* = 24) were bred on a C57BL/6 J background (Jackson Labs strain 000664; RRID:IMSR_JAX:000664) in house, exposed to 12 h of light and 12 h of dark (6 AM lights on), with chow and water provided *ad libitum*. Cx3cr1-eGFP mice (Jung et al., 2000) (Jackson Labs strain 005582; RRID:IMSR_JAX:005582) were bred to a tdTomato (Ai9) (Jackson Labs strain 007909; RRID:IMSR_JAX:007909) mouse line. Cx3cr1, the fractalkine receptor, is expressed on microglia and monocytes and hetereozygocity of this line (Cx3cr1^G/+^) allows the labeling of microglia with GFP with a haploinsufficiency for CX3CR1 (Jung et al., 2000). Ai9^+/+^/Cx3cr1^G/G^ mice were bred to a Purkinje cell specific cre line (L7-cre) (Jackson Labs strain 004146; RRID:IMSR_JAX:004146), which allows the fluorescent labeling of Purkinje cells. Together these allow the *in vivo* fluorescent imaging of microglia and Purkinje cells in the same L7^cre^/Ai9^+/−^/Cx3cr1^G/+^ mouse. Both male and female mice were used in all the experiments and analyzed for sex-specific trends. Daily monitoring of breeding pairs occurred to check for the birth of pups, considered postnatal day (P) 0.

### 2.2. Ethanol dosing

On P4, pups were toe-clipped for identification. Male and female mice were weighed and subcutaneously injected from P4 to P9 with a 5.0 g/kg/day ethanol solution (20% v/v ethanol in saline) or saline (0.9% NaCl), broken into two doses, given 2 h apart. After injection, pups were returned to the breeding cages with the dams. This is an established model used previously in our laboratory (Wong et al., 2018, 2021). Injection of only the pups minimizes confounds of reduced maternal care due to intoxication and allows for littermate controls. Mixed litters were used with 1 male and 1 female taken for each cohort (Ethanol and Saline) for a total of 4 mice per litter, when possible. Distribution across treatment groups was as follows: Ethanol, *n* = 12, six males and six females; Saline, *n* = 12, six males and six females. Animals from 13 separate litters were used, as not all litters yielded males and females. Additionally, some animals did not survive cranial window surgery or did not have window quality sufficient to image and collect data. Animals were weaned on P21. All data analysis was performed blind to exposure group.

**Figure 1:**
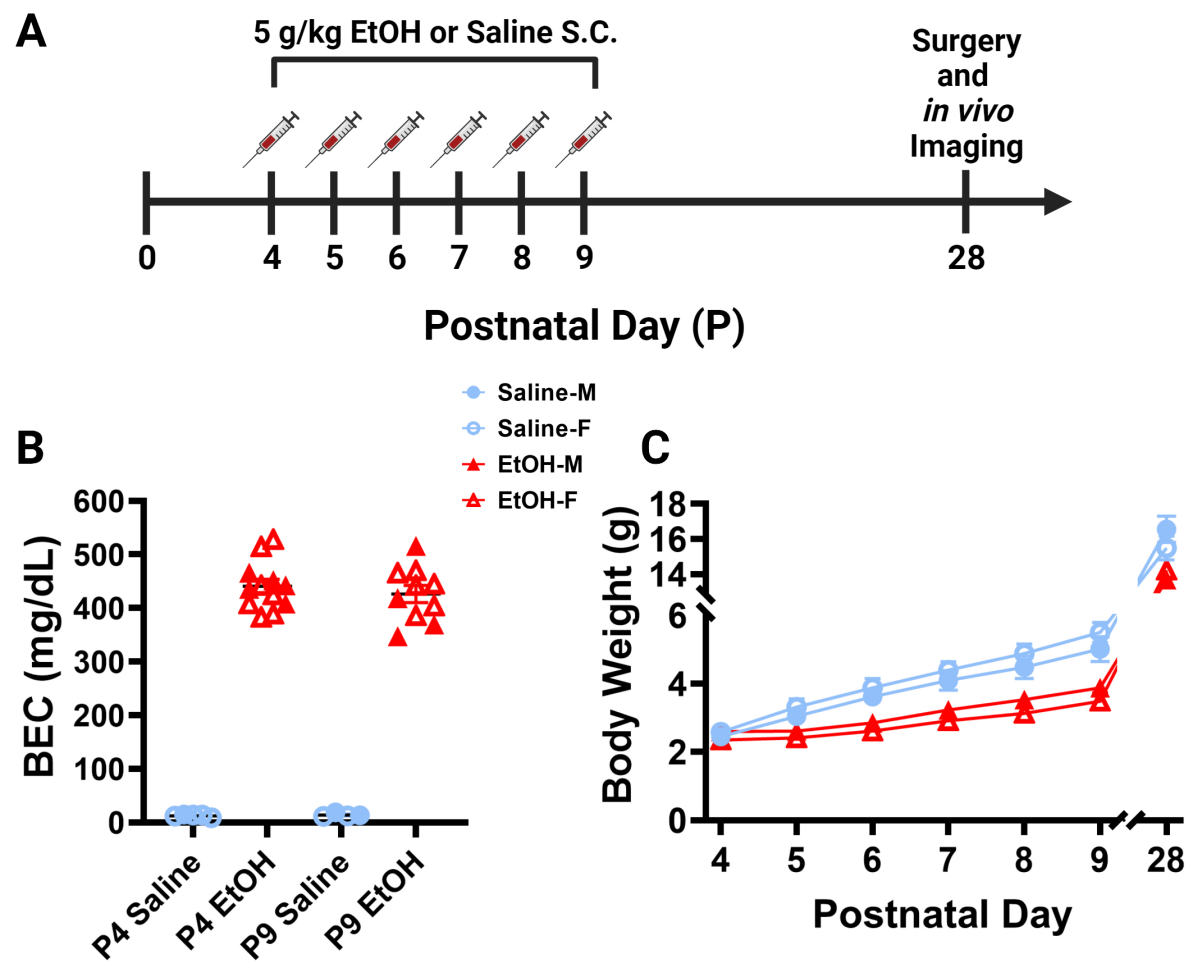
Mouse model of third trimester binge-level ethanol exposure. **(A)** Timeline of third trimester equivalent dosing paradigm. Mice were given subcutaneous injections of either 5.0 g/kg/day of EtOH or Saline, given as 2 doses, separated by 2 h each day from P4–9. Animals underwent cranial window surgery and *in vivo* two-photon imaging in adolescence around P28. **(B)** Blood EtOH Concentrations (BEC) were measured 90 min after the second dose of ethanol on either P4 [5 males (M; closed shapes), 7 females (F; open shapes) or P9 (4M, 6F)]. Pups dosed with saline were measured 90 min after the second dose as controls on P4 (1M, 4F) and P9 (2M, 2F). Individual points represent individual animals. Data are presented as the mean ± SEM. Animals dosed with EtOH displayed BECs of ~450 mg/dl on P4 and P9. **(C)** Body weights of EtOH (6M, 6F) and Saline (6M, 6F) dosed mice. Weights were collected prior to the first dose of EtOH or saline from P4–9 and prior to surgery on P28. EtOH dosed mice weighed less than their control counterparts, but all animals gained weight consistently throughout the study. There was a significant interaction between age and treatment on weight when sexes were pooled [*F*(6, 132) = 7.249, *p* < 0.0001]. There was a main effect of age [*F*(1.232, 27.11) = 1,333, *p* < 0.0001] and a main effect of treatment on body weight over time [*F*(1, 22) = 30.15, *p* < 0.0001]. Saline animals gained weight every day (*p* < 0.01) and EtOH animals gained weight every day (*p* < 0.05) except for the first day between P4 and P5 (*p* > 0.99). EtOH dosed animals had significantly lower weights than saline dosed animals every day except the first day when sexes were pooled (*p* < 0.05). Saline and EtOH dosed males had no significant differences in weight at any age, while EtOH females weighed significantly less than saline females at P6 (*p* = 0.0277), P7 (*p* = 0.0068), P8 (*p* = 0.0054), and P9 (*p* = 0.0030). **(C)** Each datapoint represents a treatment group and sex. Data are presented as the mean ± SEM. Two-way ANOVA, repeated measures with Bonferroni *post hoc* comparisons.

Separate cohorts of animals were generated for blood ethanol concentration (BEC) analysis. The 5.0 g/kg/day ethanol dosage produced BECs of ~450 mg/dl, considered a binge-level dose, in L7^cre^/Ai9^+/−^/Cx3cr1^G/+^ mice on P4 and P9 (Figure 1B). A binge-level amount of ethanol is appropriate as mice metabolize ethanol more quickly than humans, so this mimics human exposure levels more accurately (Gohlke et al., 2008; Cederbaum, 2012). For the P4 timepoint, samples were derived from 5 male and 7 female ethanol treated pups, and from 1 male and 4 female saline treated pups spread across 5 litters. For the P9 timepoint, samples were derived from 4 male and 6 female ethanol treated pups, and from 2 male and 2 female saline treated pups spread across 4 litters. Whole trunk blood was collected 90 min after the second injection on either P4 or P9 *via* decapitation. Blood was centrifuged at 4,000 rpm for 5 min at 4°C (Jouan CR3i Multifunction Centrifuge), before being stored at −80°C until it was measured using an Analox Alcohol AM1 Analyzer (Analox Technologies, Atlanta, GA, United States) (Drew et al., 2015).

### 2.3. Cranial window surgeries

L7^cre^/Ai9^+/−^/Cx3cr1^G/+^ mice given ethanol or saline from P4–9 had cranial windows implanted in adolescence (when the cerebellum is largely developed) to be used for subsequent visualization of microglia and Purkinje cells with *in vivo* two-photon imaging (Rahimi-Balaei et al., 2018). Adolescent mice between P25–31 (referred to hereafter as P28) were weighed and anesthetized with fentanyl cocktail (fentanyl 0.05 mg/kg; midazolam, 5.0 mg/kg; dexometomidine, 0.05 mg/kg) delivered intraperitoneally (i.p.). Cranial windows were implanted over lobules IV/V of the cerebellum using methods described previously (Stowell et al., 2018; Wong et al., 2018, 2021; Stowell and Majewska, 2020). In brief, aseptic technique was adhered to during surgery. Animal body temperature was maintained at 37°C and the eyes were covered with lubricant ointment during all surgical procedures and later imaging. The scalp was partially removed and membranes over the cerebellum were cleared. A 3 mm circular craniotomy was made, and the skull replaced with a glass coverslip. A headpost was fitted to the scalp and the entire fixture secured with C&B Metabond dental cement (Parkell). Cement was allowed to dry for 1 h before imaging. Surgeries and imaging were performed throughout the animals’ light phase at times dictated by equipment availability precluding controlling for circadian rhythm effects.

### 2.4. Two-photon *in vivo* imaging

Immediately after surgery and while still anesthetized, the L7^cre^/Ai9^+/−^/Cx3cr1^G/+^ mice were affixed to an imaging stage. A custom two-photon laser-scanning microscope was used for *in vivo* imaging of microglia dynamics, morphology, and Purkinje cell interactions (Ti: Sapphire, Mai-Tai, Spectra Physics; modified Fluoview confocal scan head, 20X water-immersion objective, 0.95 numerical aperture, Olympus). Excitation was achieved with 100-fs laser pulses (80 MHz) at 920 nm with a power of ~30 mW measured at the sample. A 565 dichroic with 520/40 (GFP) and 598/30 (Ai9) filters was used to visualize microglia (GFP) and Purkinje cells (Ai9) in different channels simultaneously. Imaging of 101 μm z-stacks at a 1 μm z-step size with a 4x digital zoom with an 800 × 600 pixel frame size occurred with time-lapse imaging at 5-min intervals for 1 h per imaging session. All image analysis was run offline using Ilastik (Berg et al., 2019; RRID:SCR_015246), NIH ImageJ^1^ (RRID:SCR_003070) or FIJI (see footnote 1; RRID:SCR_002285), and MATLAB (MathWorks, version R2020a; RRID:SCR_001622). Some bleed-through of the Ai9 Purkinje cell fluorescence into the background of the GFP microglia channel occurred due to spectral overlap and bleed-through correction was performed in ImageJ/FIJI as described below.

### 2.5. Image analysis

#### 2.5.1. Image preprocessing

All images were blinded using a custom MATLAB script after collection and prior to analysis.

*Bleed-through Correction*: ImageJ/FIJI was used to subtract the Ai9 Purkinje cell channel from the GFP microglia channel using a modified protocol from (Wong et al., 2021). A custom FIJI/ImageJ macro was created to automate this process. In brief, the two-channel image was split into two separate Ai9 Purkinje cell and GFP microglia channels. For each channel, the image was further split into 12 separate time points. Each channel was corrected for background fluorescence, and then a brightness compensated Ai9 Purkinje cell channel was subtracted from the GFP microglia channel. The original, raw Ai9 channel images were used for microglia-Purkinje cell interaction analysis. The final background- and Ai9-subtracted GFP microglia images were used for all microglial analyses: dynamics, morphology, and interaction.

*Denoising:* Further preprocessing was done to denoise both microglia and Purkinje cells channels. To remove high frequency components responsible for image noise, Principal component analysis (PCA) was performed and images were reconstructed using a quantitatively-determined number of components with a custom MATLAB script.

*Layer separation:* Images were divided into the Molecular Layer (ML) and Purkinje Cell Layer (PCL) of the cerebellum. This was determined manually using the Ai9 Purkinje cell channel, through the identification of the Purkinje cells somas. When the somas could be identified, this was considered the start of the Purkinje Cell Layer and the end of the Molecular Layer. Number of z slices were kept uniform and the smallest number of z slices for each layer was chosen across all animals for consistency. Substacks were created in ImageJ/FIJI and all subsequent analysis was separated by layer.

#### 2.5.2. Object classification

For automated detection of microglia and Purkinje cells, the image classification and segmentation software, Ilastik, was used. To train for pixel classification, microglia processes and somas were traced separately for both the ML and PCL, and Purkinje cell dendrites and branch points were traced separately in the ML while Purkinje cell somas were traced in the PCL. Appropriate thresholding and size exclusion criteria were applied for object classification. Microglia and Purkinje cell object output were binarized for further analysis. Microglia processes and somas, as well as Purkinje cell dendrites and branch points, were added together in ImageJ/FIJI to get whole microglia and whole Purkinje cell binary images.

#### 2.5.3. Microglia dynamics

Microglia dynamics, quantified by microglia motility and surveillance, were analyzed for ML and PCL separately. As described above, 101 μm z-stacks were collected every 5 min for 1 h for a total of 12 time points, then subdivided into the ML and PCL. ImageJ/FIJI was used to drift-correct and create max projections of each time point for each layer, leading to 24 max z-projected images for each animal (12 time points for each of the 2 layers) (previously described in Sipe et al., 2016; Whitelaw et al., 2020). These max projected images were used in Ilastik for cell detection as described above. The Ilastik binarized output files were then compared across time points using a custom MATLAB script to record microglia motility and surveillance. The motility index (M.I.) of the microglial processes was calculated by dividing the sum of the thresholded extended and retracted pixels by stable pixels between consecutive timepoints to determine how microglia sample their environment. The surveillance index (S.I.) was calculated by dividing the number of binarized microglia pixels by all pixels in the maximum projection of all timepoints to determine how much of the parenchyma microglia survey over time.

#### 2.5.4. Microglia morphology: Sholl

Microglial morphology in the first time point was assessed using Sholl analysis to quantify arbor complexity. In ImageJ/FIJI, 1–2 individual microglia were selected from the first time point of max projected images for each layer (projection creation described in microglia dynamics). The microglia processes were manually traced in ImageJ/FIJI. The ImageJ/FIJI Sholl analysis plug-in was run with concentric rings at 2 μm intervals out to 80 μm from the soma center. The number of intersections across each ring were used to create Sholl curves.

#### 2.5.5. Microglia-Purkinje cell interactions

Microglia dynamic interactions with Purkinje cells were quantified over time in 3D. For each animal, the 12 z-stacks that were subdivided into the ML and PCL as described above were placed into Ilastik for cell identification and binarization (note these were not max projected as in microglia dynamics or morphology analysis). The binarized outputs were then entered into ImageJ/FIJI for further analysis. The number of pixels were measured for whole cells and their individual components. The binarized images were multiplied together to find the overlap between microglia and Purkinje cells. The number of pixels of the resultant images were also measured. These pixel numbers were compared across conditions and over time. Interactions were analyzed separately and normalized to the sum of the microglia and Purkinje cell pixels. All 12 timepoints were averaged for analysis of condition and sex differences. Additionally, the binarized resultant images displaying the overlap of microglia and Purkinje cells were processed with the microglia dynamics analysis described above. The resultant images were drift-corrected and max projected for each time point for each interaction type in ImageJ/FIJI. The max projected, binarized resultant images were then compared across time points using the custom MATLAB script to record interaction dynamics. The interaction coverage index (similar to the S.I. described above) was calculated by dividing the number of binarized interaction pixels by all pixels in the maximum projection of all timepoints to determine how much area microglia-Purkinje cell interactions cover over time.

### 2.6. Statistics

Statistical tests were run using GraphPad Prism IX statistical analysis software (La Jolla, CA; RRID:SCR_002798), which was also used for graphing. Student’s unpaired *t*-tests were used to compared ethanol vs. saline treated animals when sexes were pooled. Two-Way ANOVAs were used to evaluate any sex differences in ethanol vs. saline treated animals with Bonferroni post-hoc comparisons used to evaluate significance where appropriate. Paired *t*-tests were used to assess layer specific changes in microglia. Detailed statistics are provided in the figure legends.

## 3. Results

### 3.1. Mouse model of third trimester binge-level ethanol exposure

To examine the later life effects of developmental ethanol exposure, we subcutaneously injected male and female L7^cre^/Ai9^+/−^/Cx3cr1^G/+^ mouse pups with binge-level amounts of ethanol (EtOH) from postnatal day (P) 4–9 (Figure 1A). This time period is the equivalent of the human third trimester and a particularly vulnerable developmental window for Purkinje cells (Goodlett and Eilers, 1997). We injected 5.0 g/kg/day ethanol solution (20% v/v EtOH in saline), administered as two separate doses of 2.5 g/kg, given 2 h apart each day. Control littermates received equivalent volumes of saline (0.9% NaCl) injections. This is considered a high binge-level amount, producing blood ethanol concentrations (BEC) of ~450 mg/dl 19 min after the second injection on either P4 or P9 (Figure 1B). This level of ethanol exposure led to lower weights of ethanol-dosed animals compared to saline controls when sexes were pooled although all animals gained weight consistently throughout the study (Figure 1C). *Post hoc* testing showed no changes between control and EtOH-dosed males and only early changes in females that equalized after the dosing paradigm was complete, suggesting that EtOH’s effect on weight gain was subtle. Subtle reductions in body weight after P4–9 ethanol exposure are consistent with previous studies (Wong et al., 2018, 2021). Pups were returned to the dams until weaning. In adolescence between P25–31, referred to as P28, mice were weighed again before undergoing cranial window surgeries (Figures 1A,C). While ethanol dosed animals weighed less than saline dosed animals at P28 when sexes were pooled, there was no significant effect of sex on body weight in either condition (Figure 1C). These surgeries allowed for immediate two-photon *in vivo* imaging of cerebellar microglia and Purkinje cells.

### 3.2. Adolescent cerebellar microglial dynamics are unaffected by binge-level developmental ethanol exposure

Using two-photon *in vivo* imaging, we examined cerebellar microglia dynamics. As highly motile cells, microglia play an important role in shaping neuronal development (Tremblay et al., 2010; Kana et al., 2019; Wong et al., 2021). Our L7^cre^/Ai9^+/−^/Cx3cr1^G/+^ mice have fluorescent microglia, which allows for the visualization of microglia dynamics, such as motility and surveillance, after cranial window surgery. For each animal, we collected 101 μm *z*-stacks every 5 min for 1 h leading to a total of 12 time points for each animal. Within each *z*-stack, we captured both the Molecular Layer (ML) that houses the dendrites of the Purkinje cells, and the Purkinje Cell Layer (PCL) that houses the Purkinje cell somas.

**Figure 2:**
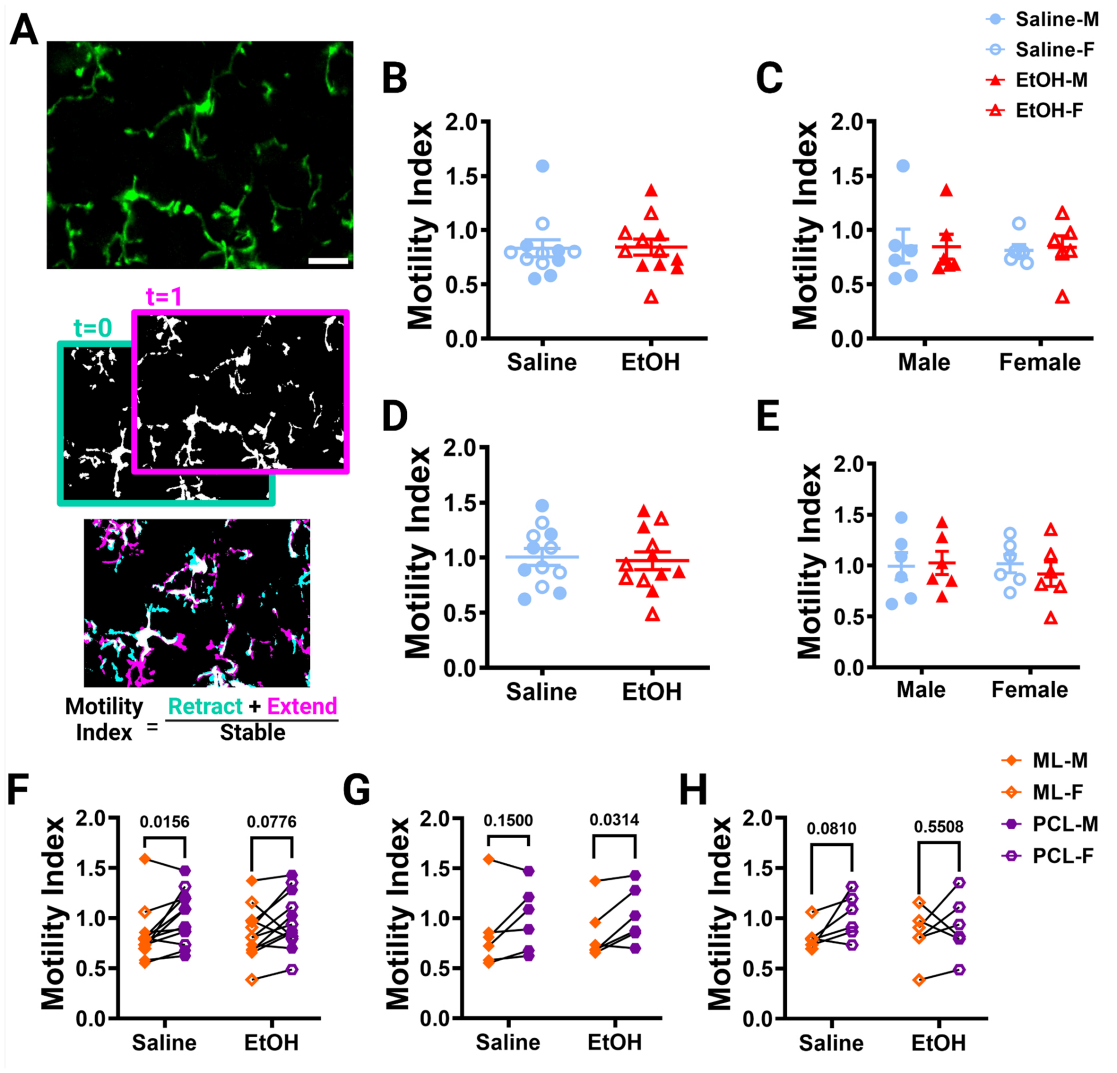
Microglia motility is unaffected by developmental ethanol exposure. **(A)** *In vivo* two-photon image of cerebellar microglia. Max z-projection of an individual timepoint (top). Binarized microglia from consecutive timepoints with time 0 in blue and time 1 in pink (middle). Overlay of the two timepoints with retracted (blue), extended (pink), and stable (white) pixels, used to create the motility index (bottom). Adolescent cerebellar microglia motility in the ML **(B,C)** and PCL **(D,E)** are unaffected by developmental ethanol exposure (triangles, 6M, 6F) compared to saline controls (circles, 6M, 6F) when sexes are pooled (**B**, ML: *p* = 0.9216; **D**, PCL: *p* = 0.7581) or examined individually **(C,E)**. There were no interactions between treatment or sex for ML [**C**, *F*(1, 20) = 0.02137, *p* = 0.8852] or PCL [**E**, *F*(1, 20) = 0.3416, *p* = 0.5654] motility. No main effects of treatment [**C**, ML: F (1, 20) = 0.009031, *p* = 0.9252; **E**, PCL: *F*(1, 20) = 0.09055, *p* = 0.7666] or sex [**C**, ML: *F*(1, 20) = 0.04172, *p* = 0.8402; **E**, PCL: *F*(1, 20) = 0.1349, *p* = 0.7173] were observed for either layer’s motility. Individual points represent individual animals with closed shapes representing males and open shapes representing females. **(F–H)** When sexes were pooled, saline dosed animals displayed more microglia motility in the PCL than the ML (**F**, *p* = 0.0156), but not when separated into males (**G**, *p* = 0.1500) and females (**H**, *p* = 0.0810). For EtOH dosed animals, PCL microglia were more motile than ML microglia only in males (**G**, *p* = 0.0314), but not in females (**H**, *p* = 0.5508) or when sexes were pooled (**F**, *p* = 0.0776). **(B-H)** Each datapoint represents an individual animal. Data are presented as the mean ± SEM. **(B,D)** Unpaired *t*-tests; **(C,E)** two-way ANOVA; **(F,G)** paired *t*-tests. Scale = 25 μm.

Using these images, we examined microglia motility, which is a measure of the combined dynamics of extension and retraction in microglial processes. We calculated the motility index (M.I.) of the microglia by dividing the sum of the binarized extended and retracted pixels by the stable pixels between consecutive timepoints (Figure 2A). We found no differences between EtOH and saline dosed animals in the ML (Figure 2B) or PCL (Figure 2D) when sexes were pooled or analyzed individually (Figures 2C,E). Similar findings have been reported in the adolescent visual and somatosensory cortices (Wong et al., 2018, 2021), suggesting that developmental ethanol exposure has limited effects on microglia motility when measured in adolescence.

**Figure 3:**
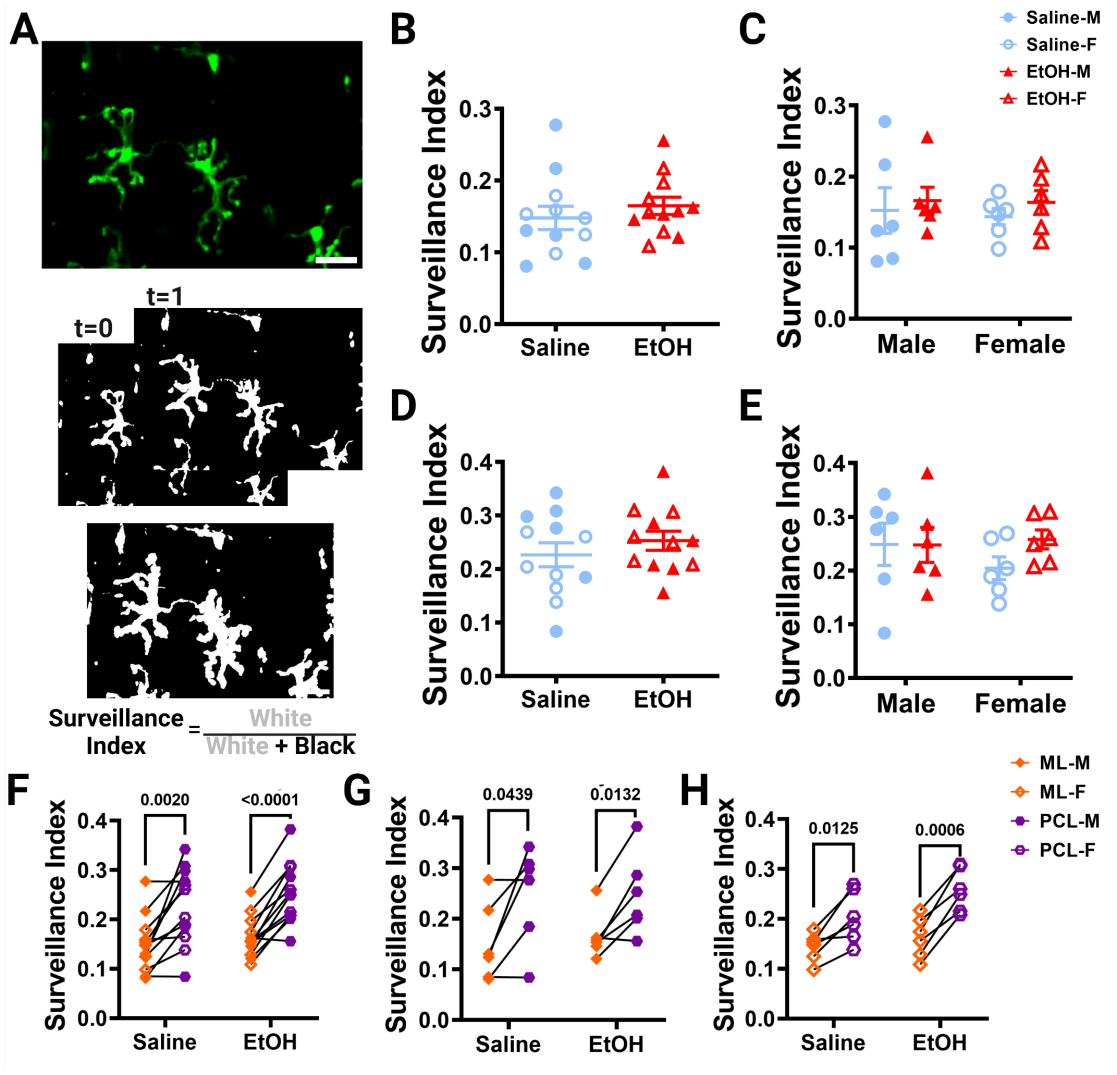
Microglia surveillance is unaffected by developmental ethanol exposure. **(A)** *In vivo* two-photon image of cerebellar microglia. Max z-projection of an individual timepoint (top). Binarized microglia from consecutive timepoints with time 0 and time 1 (middle). Overlay of all 12 timepoints with microglia (white) pixels and total (white + black) pixels, used to create the surveillance index (bottom). Adolescent cerebellar microglia surveillance in the ML **(B,C)** and PCL **(D,E)** are unaffected by developmental ethanol exposure (triangles, 6M, 6F) compared to saline controls (circles, 6M, 6F) when sexes are pooled (**B**, ML: *p* = 0.4151; **D**, PCL: *p* = 0. 0.3676) or examined individually **(C,E)**. There were no interactions between treatment or sex for ML [**C**, *F*(1, 20) = 0.02197, *p* = 0.8836] or PCL [**E**, *F*(1, 20) = 0.8901, *p* = 0.3567] surveillance. No main effects of treatment [**C**, ML: *F*(1, 20) = 0.6302, *p* = 0.4366; **E**, PCL: *F*(1, 20) = 0.8167, *p* = 0.3769] or sex [**C**, ML: *F*(1, 20) = 0.06901, *p* = 0.7955; **E**, PCL: *F*(1, 20) = 0.3459, *p* = 0.5630] were observed for either layer’s surveillance. Individual points represent individual animals with closed shapes representing males and open shapes representing females. **(F–H)** Differences in microglia surveillance were discovered between the ML and PCL in both EtOH and saline dosed animals when sexes were pooled (**F**, saline: *p* = 0.0020; EtOH: p < 0.0001), and males (**G**, saline: *p* = 0.0439; EtOH: *p* = 0.0132) and females (**H**, saline: *p* = 0.0125; EtOH: *p* = 0.0006) were analyzed individually. **(B-H)** Each datapoint represents an individual animal. Data are presented as the mean ± SEM. **(B,D)** Unpaired *t*-tests; **(C,E)** two-way ANOVA; **(F,G)** paired *t*-tests. Scale = 25 μm.

Using these same images, we also calculated the surveillance index (S.I.) by dividing the number of binarized microglia pixels by all pixels in the maximum projection of all timepoints to determine how much of the parenchyma microglia survey over time (Figure 3A). Similar to the M.I., the S.I. was unchanged in EtOH exposed animals compared to saline controls in both the ML (Figure 3B) or PCL (Figure 3D) when sexes were pooled or analyzed individually (Figures 3C,E).

Our lab previously reported layer specific differences in microglial dynamics in adult mice with PCL microglia displaying more motility than those of the ML (Stowell et al., 2018). Additionally, when mice were administered EtOH in adulthood, microglial dynamics were reduced in PCL microglia, but not ML microglia (Stowell and Majewska, 2020). We examined our mice to see if these differences were maintained in adolescent mice given EtOH postnatally. We found that PCL microglia displayed significantly more motility than those in the ML of saline-dosed animals when sexes were pooled (Figure 2F), but this significance was not maintained in either males (Figure 2G) or females (Figure 2H) individually. In EtOH-dosed animals, male mice had increased microglia motility in the PCL compared to the ML (Figure 2G), but not female (Figure 2H) or pooled (Figure 2F) mice. When we examined microglia surveillance, PCL microglia surveyed more of the parenchyma than ML microglia when sexes were pooled (Figure 3F), and when males (Figure 3G) and females (Figure 3H) were analyzed separately. This suggests that layer specific differences of microglia dynamics found in adulthood appear as early as adolescence. These differences appear to not be significantly affected by developmental EtOH exposure.

### 3.3. Adolescent cerebellar microglia morphology is unaffected by binge-level developmental ethanol exposure

**Figure 4:**
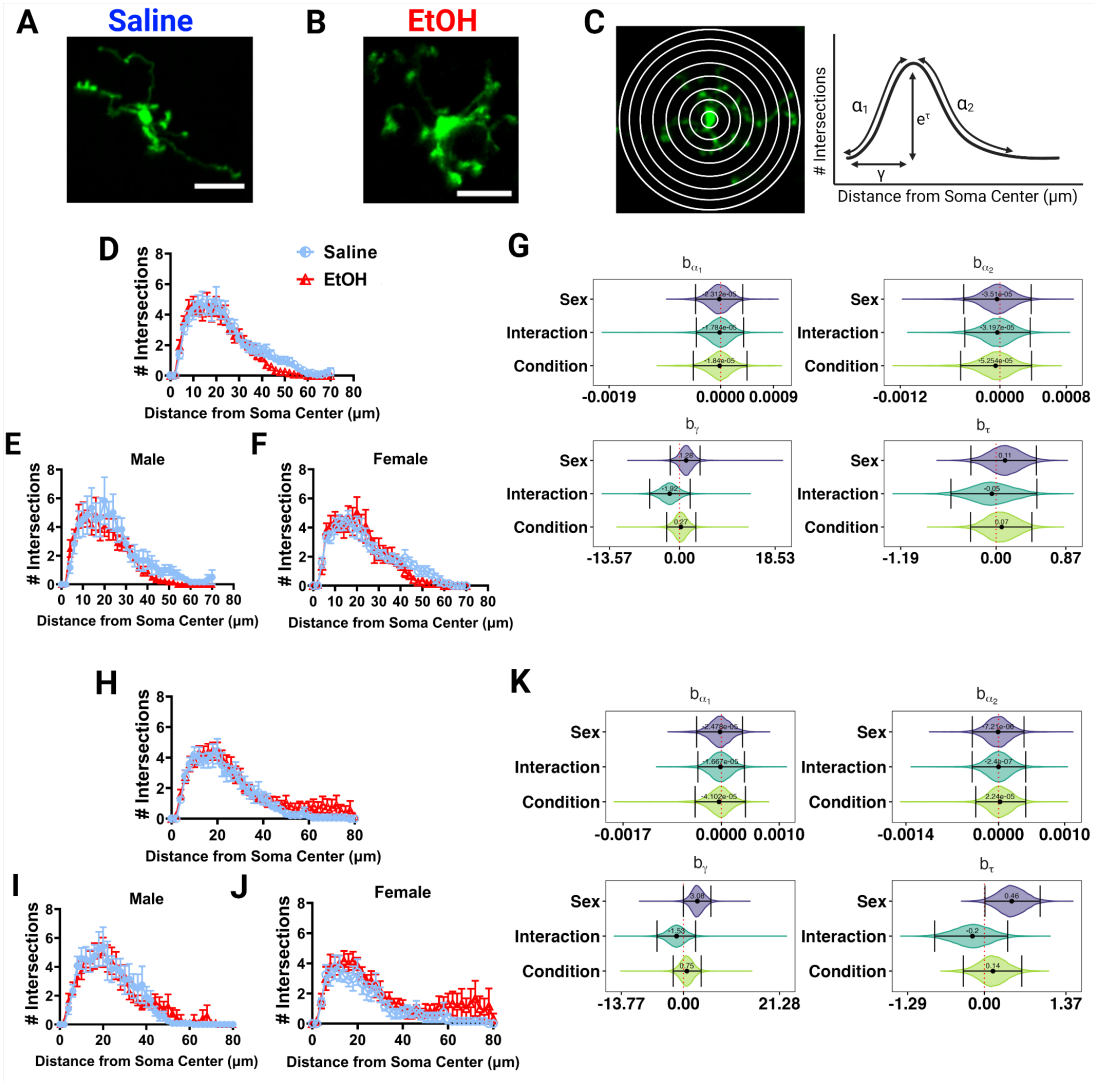
Microglia morphology is unaffected by developmental ethanol exposure. Two photon *in vivo* images of microglia in mice dosed with saline **(A)** or EtOH **(B)** from P4–9. **(C)** Two photon *in vivo* images of microglia with concentric circles drawn in increasing radii (left), which correspond to the x axis of the Sholl curves. Process intersections across these rings correspond to the y axis of the Sholl curves. Example curve (right) with labeled factors that provide information about the behavior of the Sholl curve: before the change-point (α_1_); after the change-point (α_2_); branch maximum (*e*^τ^); change-point (γ) (modified from Vonkaenel et al., 2023). Sholl curves of microglia in the ML **(D–F)** and PCL **(H–J)** show no differences in ramification between EtOH and saline groups when sexes are pooled **(D,H)**, or separated into males **(E,I)** or females **(F,J)**. In the PCL, saline male **(I)** microglia were more ramified than saline female **(J)** microglia. **(D-F, H-J)** Each datapoint represents a treatment group and sex. Data are presented as the mean ± SEM. **(G,K)** Individual Sholl curves were fit using a hierarchical Bayesian approach to capture variation at each level of the experimental hierarchy. 95% credible intervals for effects on each parameter from **(C)** were calculated across treatments and sexes for the ML **(G)** and PCL **(K)**.

Various studies have reported alterations in microglia morphology after developmental EtOH exposure, describing microglia as becoming more amoeboid and displaying hyperramification, consistent with an immune activated phenotype (Drew et al., 2015; Topper et al., 2015; Kane et al., 2021; Lowery et al., 2021). We examined *in vivo* microglia morphology in the ML and PCL of the cerebellum in these same mice (Figures 4A,B). Using the first of the 12 timepoints previously described, we traced the microglial arbors and performed Sholl analysis to examine process ramification. This analysis generates Sholl curves that plot the number of intersections of microglia processes across concentric circles of increasing radii (Figure 4C).

EtOH and saline dosed mice had similar microglial morphologies in the ML or PCL when sexes were pooled or analyzed individually (Figures 4D–F, H–J). However in the PCL, microglia in male mice seemed to have increased ramification compared to female mice (Figures 4I,J). This was confirmed with further analysis using a novel fully parametric model-based approach (Figures 4G,K; Vonkaenel et al., 2023). Effect plots show that the branch maximum (*e*^τ^) and change point (γ) were modulated with sex but not treatment in the PCL for saline, but not for EtOH dosed animals (Figure 4K). Limited effects of developmental ethanol exposure on microglia morphology have also been reported in the adolescent visual and somatosensory cortices (Wong et al., 2018, 2021). Ethanol administration in adulthood similarly did not affect ML microglia morphology (Stowell and Majewska, 2020). This suggests that developmental ethanol exposure does not have a large effect on cerebellar microglia morphology when assessed later in life.

### 3.4. Adolescent cerebellar microglia-Purkinje cell interactions may be affected by binge-level developmental ethanol exposure

**Figure 5:**
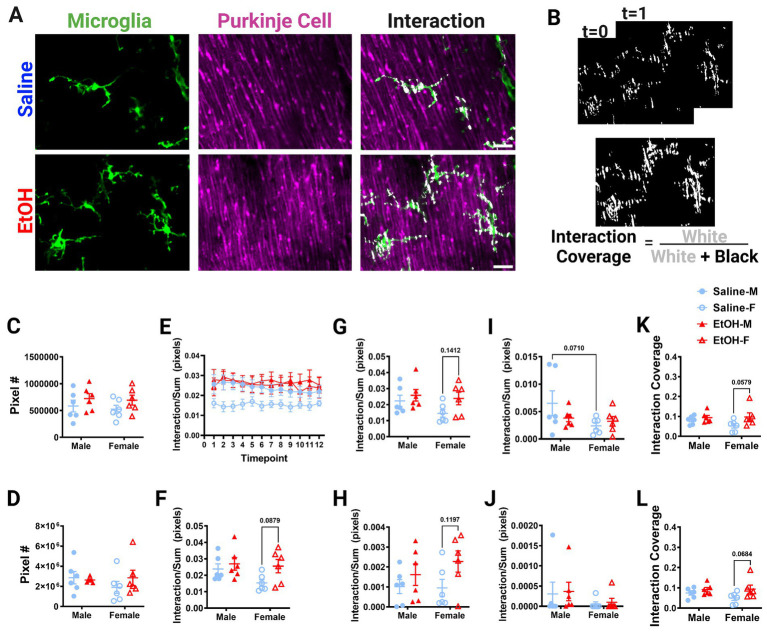
Molecular Layer cerebellar microglia-Purkinje cell interactions are relatively unaffected by ethanol. **(A)** Two-photon *in vivo* images of microglia (left), Purkinje cells (middle), and their interactions (right) when mice were given saline (top) or EtOH (bottom) from P4–9. The white overlay in the interaction images indicate the overlap between cells. **(B)** Binarized interactions from consecutive timepoints with time 0 and time 1 (top) from EtOH animal shown in **(A)**. Overlay of all 12 timepoints with interaction (white) pixels and total (white + black) pixels, used to create the interaction coverage (bottom). **(C)** Whole microglia (microglia somas and processes) pixel numbers were unaffected by treatment [*F*(1, 20) = 2.790, *p* = 0.1104]. **(D)** Whole Purkinje cell (branch points of dendrites and non-branch point areas of dendrites) pixel numbers were unaffected by treatment [*F*(1, 20) = 0.3868, *p* = 0.5410]. **(E)** Whole microglia-whole Purkinje cell interactions normalized to total pixels representing microglia (MG) and Purkinje cell (PC) pixels over the 12 timepoints. **(F–J)** Comparisons of microglia-Purkinje cell interactions normalized to the sum of MG and PC pixels when all timepoints are averaged together for the whole MG (processes + somas) and whole PC (dendrites + branchpoints) **(F)**; MG processes and PC dendrites **(G)**; MG somas and PC dendrites **(H)**; MG processes and PC branchpoints **(I)**; MG somas and PC branchpoints **(J)**. While none were statistically significant, some trends toward increased interactions in EtOH mice compared to saline mice were observed: **(F)** whole MG interactions with whole PC [*F*(1, 20) = 3.951, *p* = 0.0607], driven by EtOH females (*p* = 0.0879); **(G)** MG process × PC dendrite [*F*(1, 20) = 3.477, *p* = 0.0770] driven by EtOH females (*p* = 0.1412); **(H)** MG soma × PC dendrite [*F*(1, 20) = 4.186, *p* = 0.0541], driven by EtOH females (*p* = 0.1197). Additionally sex differences were observed with a trend toward increased interactions in males compared to females in **(I)** MG process × PC branch [*F*(1,20) = 3.313, *p* = 0.0838], driven by saline males (*p* = 0.0710), but interactions were unaffected by treatment [*F*(1, 20) = 0.5150, *p* = 0.4813]. **(J)** No differences were found in MG soma × PC branch interactions [*F*(1, 20) = 0.07120, *p* = 0.7923]. **(K,L)** Dynamic interactions in EtOH animals, particularly females, covered a significantly larger area than in saline animals for both the whole MG (processes × somas) and whole PC (dendrites × branchpoints) [**K**, *F*(1, 20) = 4.513, *p* = 0.0463, Bonferroni *post hoc p* = 0.0579] and MG processes and PC dendrites [**L**, *F*(1, 20) = 4.882, *p* = 0.0390, Bonferroni *post hoc* 0.0684]. **(C,D, F–L)** Each datapoint represents an individual animal. Data are presented as the mean ± SEM. Two-way ANOVA with Bonferroni *post hoc* comparisons. **(E)** Each datapoint represents a treatment group and sex. Data are presented as the mean ± SEM. Scale = 25 μm.

**Figure 6:**
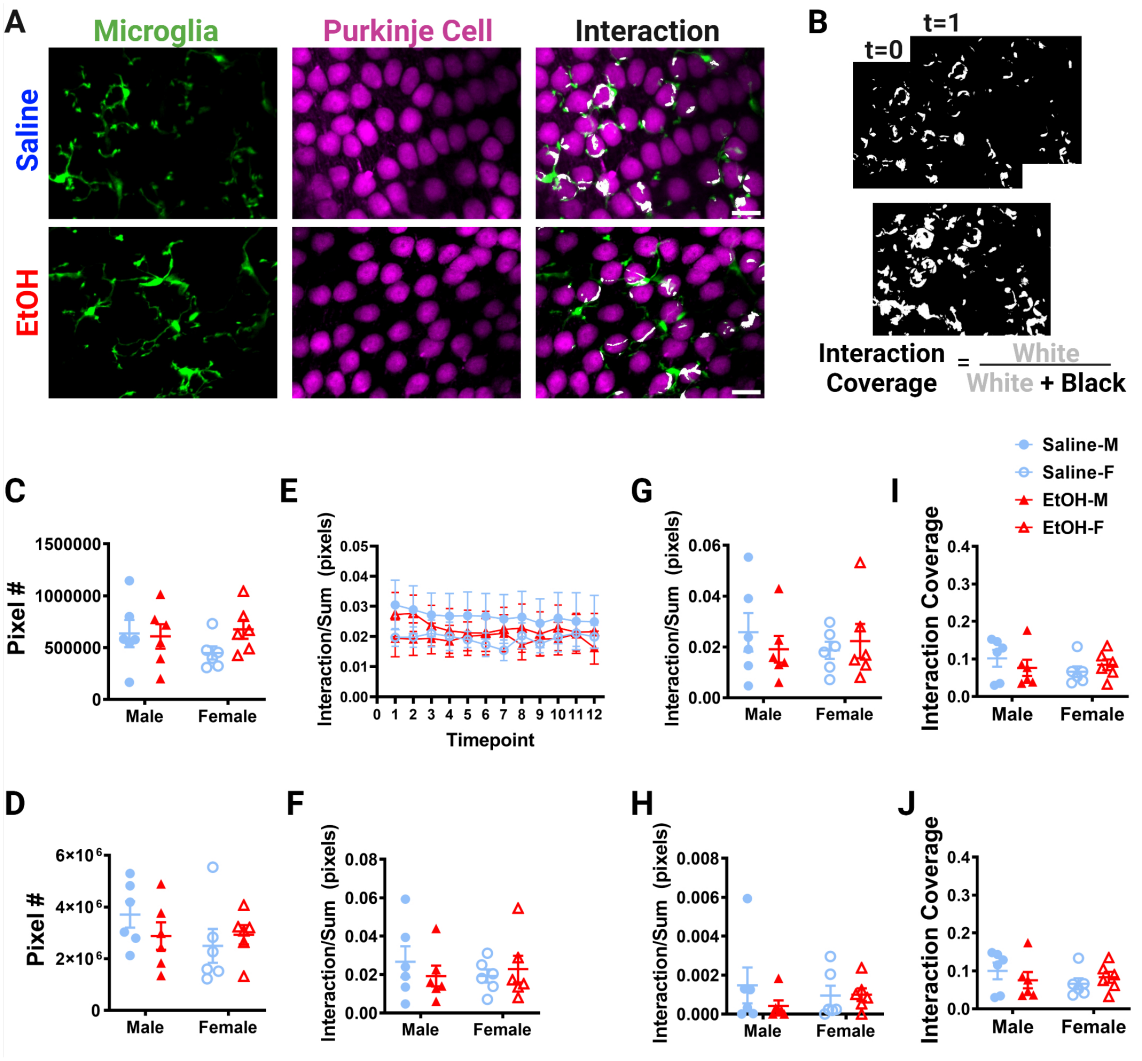
Purkinje cell layer cerebellar microglia-Purkinje cell interactions are unaffected by ethanol. **(A)** Two-photon *in vivo* images of microglia (left), Purkinje cells (middle), and their interactions (right) when mice were given saline (top) or EtOH (bottom) from P4–9. The white overlay in the interaction images indicate the overlap between cells. **(B)** Binarized interactions from consecutive timepoints with time 0 and time 1 (top) from saline animal shown in **(A)**. Overlay of all 12 timepoints with interaction (white) pixels and total (white + black) pixels, used to create the interaction coverage (bottom). **(C)** Whole microglia (microglia somas and processes) pixel numbers were unaffected by treatment [*F*(1, 20) = 0.9211, *p* = 0.3487]. **(D)** Purkinje cell soma pixel numbers were unaffected by treatment [*F*(1, 20) = 0.1463, *p* = 0.7062]. **(E)** Whole microglia-Purkinje cell soma interactions normalized to the sum of microglia (MG) and Purkinje cell (PC) pixels over the 12 timepoints for the whole MG (processes + somas) and PC somas. **(F–H)** Comparisons of microglia-Purkinje cell interactions normalized to the sum of MG and PC pixels when all timepoints are averaged together for the whole MG (processes + somas) and PC somas **(F)**; MG processes and PC somas **(G)**; MG somas and PC somas **(H)**. No differences were observed between mice given EtOH or saline in development in either male or female mice in whole MG x PC soma [**F**, *F*(1, 20) = 0.09261, *p* = 0.7640], MG process × PC soma [**G**, *F*(1, 20) = 0.06558, *p* = 0.8005], or MG soma x PC soma [**H**, *F*(1, 20) = 0.7771, *p* = 0.3885]. **(I,J)** Similarly, dynamic interaction coverage was unaffected by EtOH in the whole MG and PC soma [**I**, *F*(1, 20) = 0.03629, *p* = 0.8508] and MG process and PC soma [**J**, *F*(1, 20) = 0.03770, *p* = 0.8480]. **(C,D,F–J)** Each datapoint represents an individual animal. Data are presented as the mean ± SEM. Two-way ANOVA. **(E)** Each datapoint represents a treatment group and sex. Data are presented as the mean ± SEM. Scale = 25 μm.

While we found no differences in *in vivo* microglia dynamics or morphology, developmental ethanol exposure could affect how microglia interact with neurons. In the healthy brain, microglia play an important role in the development of neurons and connectivity by making physical contacts with neuronal somas, dendrites, and axons (Dean et al., 2012; Nakayama et al., 2018; Kana et al., 2019; Perez-Pouchoulen et al., 2019; Yamamoto et al., 2019; Cserép et al., 2021). Here, we characterized putative physical contacts between microglia and Purkinje cells in three dimensions over the 12 time points that encompass our imaging sessions in both the ML (Figure 5) and PCL (Figure 6).

Putative physical interactions were defined as areas that overlapped in signal between the microglia and Purkinje cell channels within each z plane (Figures 5A, 6A). To get further insight into the subcellular localization of interactions between microglia and Purkinje cells, we subdivided each cell into components (Supplementary Figure S1). For each layer, we further analyzed interactions with microglial somas or processes separately (as well as the whole microglia which included soma and processes). For the ML, Purkinje cell dendrites were divided into dendritic branch points (Stowell et al., 2018) or dendritic stretches outside of branch points, as well as the whole dendrite which included both types of structures. For the PCL, only the Purkinje cell somas were characterized. Various combinations of the above listed components were examined for interactions and no differences were observed (Supplementary Figure S2). The number of pixels occupied by microglia (Figure 5C, 6C) and Purkinje cells (Figures 5D, 6D) and their various sub-components (Supplementary Figure S1) were generally unchanged between sexes and conditions, except for the ML microglia soma, which had a main effect of treatment (Supplementary Figure S1), driven by increases in pixel numbers of EtOH females compared to saline females. While only one subcomponent was altered by EtOH, we normalized the value of these interactions to the sum of the microglia and Purkinje cell pixels to account for variations in microglia and Purkinje cell availability in each image (Figures 5E–J, 6E–H). In general, interactions were relatively stable over time for all conditions, sexes, and cells (components) in both the ML (Figure 5E) and PCL (Figure 6E), and averaged values across all 12 time points were used for comparisons (Figures 5F–J, 6F–H).

Developmental EtOH exposure may have mild effects on microglia-Purkinje cell interactions in the ML (Figure 5). Increased interactions in EtOH dosed mice compared to saline mice were observed for whole microglia interactions with whole Purkinje cells (Figure 5F), although these did not reach significance. Bonferroni post-hoc comparisons suggest this difference was driven by EtOH females having more interactions than saline females. We next wanted to see if these effects persisted in interactions between subcellular components. As microglial processes compose the majority of microglia, and in the ML, dendrites (without branch points) are the majority of Purkinje cells, we first examined these interactions as they would be the most likely drivers of the effect (Supplementary Figure S1). As expected, similar effects to the whole microglia and whole Purkinje cell were observed when limiting the analysis to just the microglial processes (excluding any contacts made with microglial somas) and non-branch areas of Purkinje cell dendrites (Figure 5G), with female EtOH mice having more interactions than female saline mice. Next, we examined the same non-branch areas of dendrites, but with microglia somas. Similar to their interactions with microglial processes, there was a trend to increased interactions when considering only microglial somas (no processes) and non-branch areas of Purkinje cell dendrites (Figure 5H), with female EtOH mice having more interactions than female saline mice. This suggests that EtOH may have similar effects on all microglial subcellular components when they interact with Purkinje cells’ non-branch areas of dendrites.

We next examined branch points of Purkinje cells and their interactions with microglia components. While microglial processes’ interactions with Purkinje cell dendrite branch points were unaffected by treatment (Figure 5I), there appeared to be a trend toward increased interactions in males compared to females (Figure 5I). Bonferroni post-hoc comparisons suggest this difference was driven by saline dosed animals. In the ML, microglia somas had no differences in interactions with branch areas of Purkinje dendrites in EtOH or saline mice (Figure 5J). This suggests that EtOH’s effects may be limited to interactions with non-branch areas of Purkinje cell dendrites. This may be driven by the small size of branch areas of Purkinje cell dendrites, so there may be fewer opportunities for interactions with these subcellular components (Supplementary Figure S1). To determine the dynamics of microglia-Purkinje cell interactions, we performed a coverage analysis where we overlaid all areas of interaction across 12 time points in two dimensions for both the ML (Figure 5B) and PCL (Figure 6B). This allowed us to compare the cumulative interactions over the 1 h imaging period. We found that dynamic interactions between whole microglia and whole Purkinje cells covered a larger area in EtOH dosed animals (Figure 5K), an effect driven by increases in females. A similar effect was observed when coverage was computed for microglia process interactions with non-branch areas of Purkinje cells dendrites (Figure 5L), also driven by females.

No differences were noted between EtOH and saline dosed mice or between males and females in the PCL in whole microglia interactions with Purkinje cell somas (Figure 6F), microglia process interactions with Purkinje cell somas (Figure 6G), or microglia soma interactions with Purkinje cell somas (Figure 6H). Similarly, the dynamic coverage of microglia-Purkinje cell soma interactions was unaffected by EtOH for whole microglia and Purkinje cell soma interactions (Figure 6I), as well as microglia process and Purkinje cell soma interactions (Figure 6J). This suggests EtOH may affect microglia interactions with neurons later in life in a layer specific manner.

## 4. Discussion

While much work has been completed on microglia and Purkinje cells individually after developmental ethanol exposure, little is known about their interactions. Adolescent microglia dynamics in mice exposed to ethanol in development have been examined in the cortex but have thus far been unstudied in the cerebellum (Wong et al., 2018, 2021). As microglia display regional heterogeneity in the absence of perturbation (Grabert et al., 2016; Stowell et al., 2018) as well as after ethanol exposure (Drew et al., 2015), developmental ethanol exposure may have differing effects on cerebellar microglia dynamics compared with cortical microglia dynamics. A more immune alert state found in cerebellar microglia may translate to greater alterations in these cells after an insult or in disease compared to cortical microglia (Grabert et al., 2016). Additionally, while microglia-Purkinje cell interactions have been examined in the adult mouse, no work has examined these interactions in adolescence or after ethanol exposure (Stowell et al., 2018). Understanding the development of these interactions may be important for understanding FASD pathology and critical windows of development. At the time of our study, around four postnatal weeks of age, the cerebellum is mature (Rahimi-Balaei et al., 2018). Our P4–9 ethanol dosing model occurs during a critical period of development for the cerebellum and Purkinje cells (Goodlett et al., 1990; Hamre and West, 1993; Goodlett and Eilers, 1997; Topper et al., 2015). Microglia-Purkinje cell interactions may be altered long-term by developmental EtOH exposure, and these alterations may contribute to FASD pathology.

In this work, we examine the effects of a binge-level exposure of ethanol on a mouse model in the human third trimester equivalent on cerebellar microglia and Purkinje cells. We sought to determine if microglia dynamics are affected later in life when mice are given ethanol in development. We found no changes in cerebellar microglia motility or surveillance in either the ML or PCL when mice were given ethanol compared to saline. However, PCL microglia were more motile and displayed more surveillance compared to ML microglia. While there were no differences in arbor complexity between EtOH and saline mice in the ML, male mice microglia were more ramified than female mice microglia in the PCL in saline dosed mice. Additionally, while most of our data did not reach statistical significance, we found trends toward possible increases in microglia-Purkinje cell interactions in female mice in the ML, but not the PCL, after developmental EtOH exposure.

Our finding of unchanged microglia dynamics after developmental ethanol exposure is in line with other work that has reported no effect of developmental ethanol exposure in similarly aged mice on cortical microglia dynamics (Wong et al., 2018, 2021). While EtOH reduced microglia motility and surveillance in the cerebellum in adult mice, in that study EtOH was given acutely in adulthood immediately prior to imaging, instead of in development (Stowell and Majewska, 2020). EtOH may have similar effects on motility during an early exposure, but the weeks of time between the last EtOH exposure in our study and imaging may have been enough time for resolution of any ethanol induced effects and return to homeostasis. Examining the cerebellum immediately after the last ethanol exposure on P10 may be more likely to yield differences. Indeed, examination of the cerebellum and hippocampus at P10 after a similar developmental exposure uncovered increases in the proinflammatory factors, IL-1β, TNF-α, and CCL2 (Drew et al., 2015). Such effects of developmental ethanol exposure have been found to be transient in some cases. An acute, binge-level dose of EtOH in mice on P7 or P8 led to microglia displaying a more amoeboid phenotype and increases in pro-inflammatory cytokines, a phenotype that resolved within 24–48 h after exposure (Ahlers et al., 2015). Additionally, we examined microglial homeostatic functions, but not the immune function of these microglia, where an immune “second-hit” may be needed to uncover effects of developmental ethanol exposure later in life. In the cortex, adolescent microglia trended toward a faster response to a laser ablation injury when given ethanol in development compared to controls (Wong et al., 2018, 2021). A similar insult later in life could uncover long-lasting effects on cerebellar microglia that were not evident in this study, where we focused on microglial dynamics in the absence of additional insults. Future experiments to examine how developmentally exposed cerebellar microglia respond to immune challenge would be valuable to help us understand whether these cells’ activity is altered by ethanol.

We also did not find ethanol-induced changes in microglial morphology. Developmental ethanol exposure has been known to increase amoeboid morphology and alter ramification (Ahlers et al., 2015; Topper et al., 2015), and a single binge-level exposure to ethanol can lead to subtle changes in glia when examined 24 h later (Lowery et al., 2021). While this could also be due to the time elapsed between exposure and our morphology assessment, another discrepancy may be that other studies examined microglia in fixed tissue, while we examined microglia *in vivo*. As we needed to image at sufficient magnification to capture microglia arbors, we were only able to visualize a few microglia in our field of view with two-photon imaging. During Sholl analysis, we only included microglia whose processes and somas fell within our image, so we were only able to include 1–2 microglia for each layer in each animal. The low number of microglia analyzed may mask any significant differences in EtOH versus saline mice. Additionally, a lack of morphological change does not necessarily mean that microglial function is unaltered, as microglia states are dynamic and structural changes cannot always be equated with functional ones (Paolicelli et al., 2022). This made it all the more important that we investigate other aspects of microglia function, such as their dynamic interactions with neurons that could inform on altered microglial function, as well as possible effects on neurons (Whitelaw et al., 2022).

Microglia are known to have dynamic interactions with neurons, both in the cortex and cerebellum (Wake et al., 2009; Tremblay et al., 2010, 2011; Stowell et al., 2018, 2019). In the cortex, microglia contact synapses and modulate dendritic spine formation and elimination in an activity and context-dependent manner (Tremblay et al., 2010; Sipe et al., 2016). In both the healthy brain and disease, microglia are known to phagocytose neurons and prune synapses (Marin-Teva et al., 2004; Schafer et al., 2012; Ayata et al., 2018), and both the complement system and fractalkine signaling have been implicated in microglia–neuron interactions and brain plasticity (Hoshiko et al., 2012; Schafer et al., 2012). While microglia motility may be unaltered by ethanol, changes in activity or the release of factors by either microglia or neurons may still affect their interactions, as microglia can release a number of soluble factors that modulate neuronal function but do not require physical proximity. Much of the work on microglia–neurons interactions has focused on the cortex, and the study of the effects of developmental ethanol exposure on microglia-Purkinje cell interactions is novel and has thus far not been characterized. While one study previously examined these interactions *in vivo*, this occurred in adult unperturbed mice (Stowell et al., 2018). Here, we develop new analyses to interrogate the interactions between microglial components and the components of Purkinje cells. Our work suggests EtOH may lead to layer specific alterations in microglia contact with neurons. Independently of the effects of ethanol, this work gives us new understanding of the interactions between microglia and Purkinje cells in adolescence and can open new pathways to understanding how microglia physically contact neurons. Our work here only characterized putative physical interactions, because light microscopy cannot resolve direct contact between membranes. In the future, electron microscopy should be used for higher resolution to differentiate between close proximity and contacts between the cells. Microglia are known to release factors after developmental ethanol exposure that lead to inflammation, which can also affect neurons (Drew et al., 2015; Kane et al., 2021). Additionally, signaling pathways may be disrupted, which could further impact microglia–neuron interactions, and such mechanisms remain to be investigated in the context of EtOH exposure (Hoshiko et al., 2012; Schafer et al., 2012).

While microglia dynamics, morphology, and interactions were minimally affected by developmental ethanol exposure, throughout this study, we found layer specific differences in microglia. The cerebellum’s cytoarchitecture may underlie these layer specific differences. We examined the ML, which houses the dendrites of the Purkinje cells, and the PCL, which houses their somas. At the time of our imaging sessions, the PCL is a monolayer while the ML is densely packed with the Purkinje cells’ unique, elaborate dendrite arbor. We found that microglial dynamics were generally increased in the PCL compared to the ML in both saline and EtOH dosed animals, consistent with previous findings (Stowell et al., 2018). Increased coverage of microglia, measured through surveillance, in the PCL may result from the fact that the PCL houses mainly Purkinje cell and Bergmann glia somas, while the ML is filled with Purkinje cell dendrites, Bergmann glia fibers, parallel fibers of granule cells, basket and stellate cells. There may be more physical space for microglia to occupy in the PCL, which could provide more room for movement. The differences in number and type of cellular components between layers may also underlie the potential effects we observed for microglia-Purkinje cell interactions. Trends toward significant differences in microglia-Purkinje cell interactions were found between treatment and sex in the ML, but not the PCL. As the ML contains the Purkinje cell dendrites where most synapses occur, microglia may be interacting with synapses and potentially increasing pruning in EtOH dosed animals. This is consistent with the potential of increased interactions occurring in EtOH dosed animals compared to saline dosed animals in the ML, but not the PCL. It would be interesting to explore this further with future higher-resolution analysis to determine if these changes are occurring at synapses.

In addition to layer specific differences, we wanted to examine if any sex differences were observed. Current studies have not described many sex differences in the normally developing cerebellum. In one study, calbindin, a marker for Purkinje cells, was found to have higher levels of mRNA and protein in female mice than male mice around the third postnatal week (Abel et al., 2011). While we found no differences in pixel number of Purkinje cells’ dendrites or somas in either the ML or PCL, differences in calbindin expression may not result from alterations in the number or complexity of Purkinje cells. While our measures of Purkinje cells were unchanged, we also examined microglia as sex differences in microglia density, morphology, and migration have been reported (Han et al., 2021). Indeed for morphology, sex differences were found in the PCL, but not in the ML, in saline but not the EtOH dosed animals, with male mice having more ramified microglia than female mice. In the ML, saline dosed male mice trended toward more microglia process interactions with Purkinje cell branch points than saline dosed female mice, which may reflect sex-differences in microglia–neuron interactions in the cerebellum. Despite this, effects of developmental EtOH on these interactions were driven by female mice. In the ML, increases in EtOH interactions between whole microglia and whole Purkinje cells, as well as Purkinje cell non-branch areas of dendrites with either microglia processes and somas, appeared to be driven by females. When microglia subcellular components were examined, microglia soma only pixel numbers in the ML were significantly increased in EtOH females compared to saline females. The lack of differences in males is interesting, as males are known to be at a higher risk for some neurodevelopmental disorders, such as Autism Spectrum Disorder (Roux et al., 2019). FASD diagnosis varies with age and sex, with higher FASD prevalence in males than females at young ages, but unaltered diagnosis rates in older ages (Terasaki et al., 2016). If this is due to deficits appearing later in life in females, this would be consistent with our results as we examined the effects of EtOH in adolescence.

While we found minimal effects of developmental ethanol exposure on cerebellar microglia and Purkinje cells, other avenues can be explored as well, such as molecular changes. Brain region-specific differences may be observed from similar levels of EtOH exposure, so examining other regions using the same dosage and administration method may show differential effects of ethanol on microglia–neuron interactions. We quantified one type of glia–neuron interaction, but it would be interesting to explore the *in vivo* effects of ethanol on Bergmann glia and their interactions with microglia or Purkinje cells. Microglia and astrocytes both release factors and influence neuronal development and circuitry (Stogsdill and Eroglu, 2017). In the cortex and hippocampus, microglia and astrocyte contacts have been characterized after developmental ethanol exposure (Lowery et al., 2021). Bergmann glia development also occurs during the early postnatal period and these cells sheath and regulate Purkinje cell synapses (Yamada et al., 2000; Lippman Bell et al., 2010). Reduced numbers of Bergmann glia and contacts between their fibers and Purkinje cell dendrites in the ML have been reported after ethanol (Shetty and Phillips, 1992; Shetty et al., 1994; González-Burgos and Alejandre-Gómez, 2005; Yang et al., 2014). If Bergmann glia development and regulation of synapses is affected by developmental ethanol exposure, altered excitability may occur in the cerebellum, leading to some of the cognitive and behavioral deficits found in FASD.

Overall, this work suggests that developmental EtOH exposure has minimal effects on microglia dynamics, morphology, or cell–cell physical interactions with Purkinje cells in adolescence. While the effect was not significant, we describe subtle differences in microglia-Purkinje cell interactions which may have an effect on synapses and the cerebellar circuit in adulthood contributing to EtOH-induced dysfunction. Additionally, our work suggests layer and sex specific differences which should be taken into account when studying the function and dysfunction of the cerebellum.

## Data availability statement

The raw data supporting the conclusions of this article will be made available by the authors, without undue reservation.

## Ethics statement

The animal study was reviewed and approved by University of Rochester Committee on Animal Resources.

## Author contributions

MC, PD, and AM conceived the project and designed the experiments. MC and JD contributed to data collection. MC performed the experiments, imaging, and analysis for all figures. JD assisted with BEC analysis for Figure 1. LL developed some of the protocols and codes used in Figures 2–6. EV and MM assisted with the analysis and also helped interpret data presented in Figure 4. MC and AM wrote the first draft of the manuscript. All authors contributed to the final manuscript. All authors contributed to the article and approved the submitted version.

## Funding

This study was supported by the NIH Fellowship F31AA030445 (MC), Grant from the NIAAA: RO1 AA027111 (AM and PD), T32 Training Grant T32NS115705 (MC), and University of Rochester Intellectual and Developmental Disabilities Research Center (UR-IDDRC HD103536).

## Conflict of interest

The authors declare that the research was conducted in the absence of any commercial or financial relationships that could be construed as a potential conflict of interest.

## Publisher’s note

All claims expressed in this article are solely those of the authors and do not necessarily represent those of their affiliated organizations, or those of the publisher, the editors and the reviewers. Any product that may be evaluated in this article, or claim that may be made by its manufacturer, is not guaranteed or endorsed by the publisher.

## Footnotes

^1^ imagej.net
