# Supplementary material for: Developmental ethanol exposure has minimal impact on cerebellar microglial dynamics, morphology, and interactions with Purkinje cells during adolescence
Source: Front Neurosci. 2023 May 5;17:1176581. doi: 10.3389/fnins.2023.1176581 (PMC10198441; doi:10.3389/fnins.2023.1176581)
Supplement: Supplementary file 1 [file Data_Sheet_1.PDF]

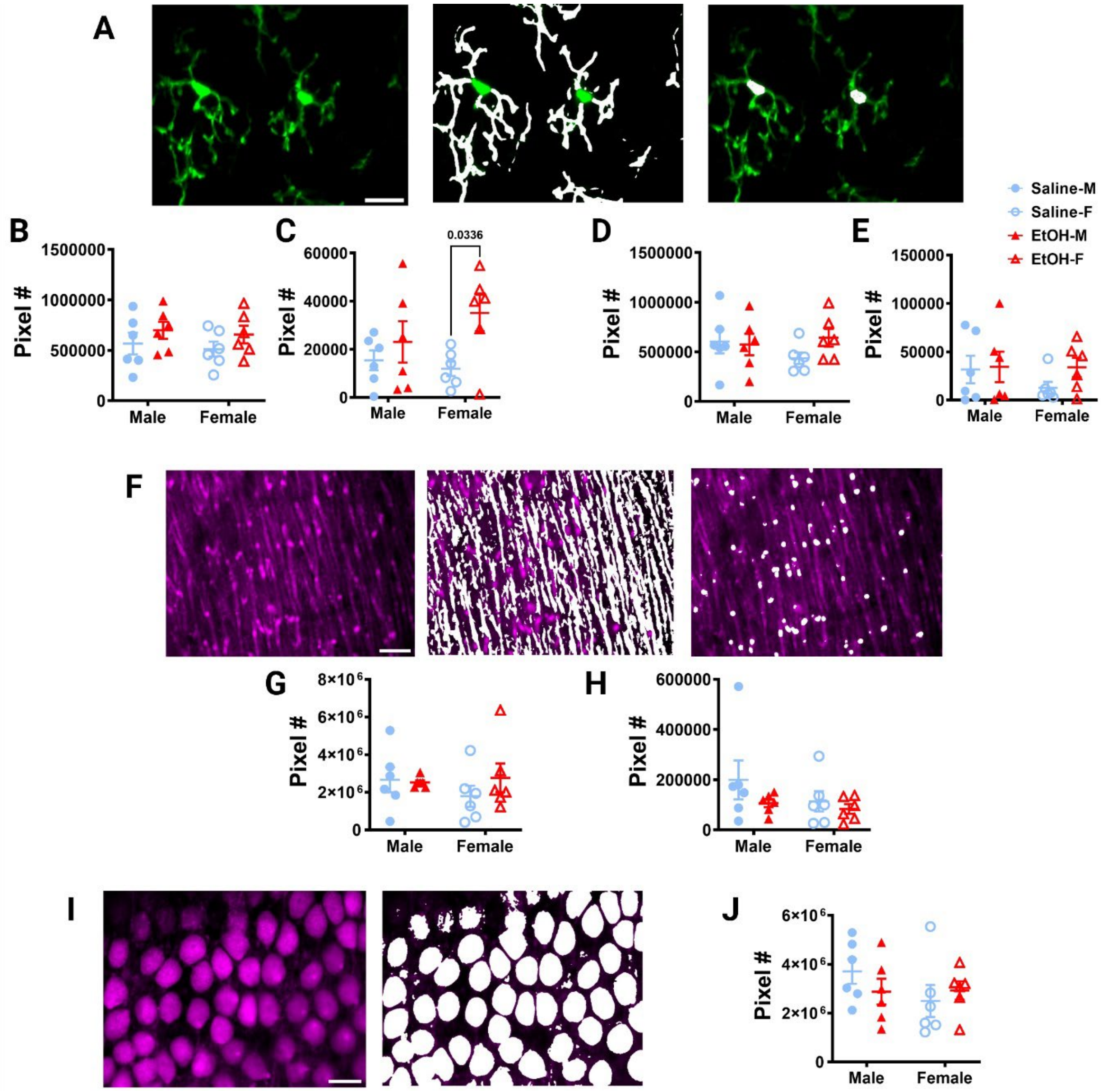

Supplementary Figure 1: Subcellular components of microglia and Purkinje cells in the ML and PCL (A) Two-photon *in vivo* images of microglia. The white overlays indicate areas characterized as microglial processes (middle) or microglial somas (right). **(B-E)** Comparisons of microglia pixel numbers when all timepoints are averaged together for microglia processes **(B, D)** or somas **(C, E)** in the ML **(B-C)** or PCL **(D-E)** were largely unaffected by treatment in male and female mice. **(C)** However, in the ML, microglia soma pixel numbers were significantly increased in EtOH animals compared to controls ($F(1, 20) = 6.025$, $P=0.0234$), driven by EtOH females ($P=0.0336$). **(F)** Two-photon *in vivo* images of Purkinje cell dendrites, including branch points, in the ML. The white overlays indicate areas characterized as Purkinje cell non-branch areas of dendrites (middle) or branch points (right). **(G-H)** Pixel numbers of Purkinje cell non-branch areas of dendrites **(G)** or branch points **(H)** were unaffected by treatment. **(I)** Two-photon *in vivo* images of Purkinje cell somas in the PCL. The white overlay indicates areas characterized as Purkinje cell somas. **(J)** Pixel numbers of Purkinje cell somas were unaffected by treatment. **B-E, G-H, J:** Two-way ANOVAs with Bonferroni post-hoc comparisons. Scale= 25µm

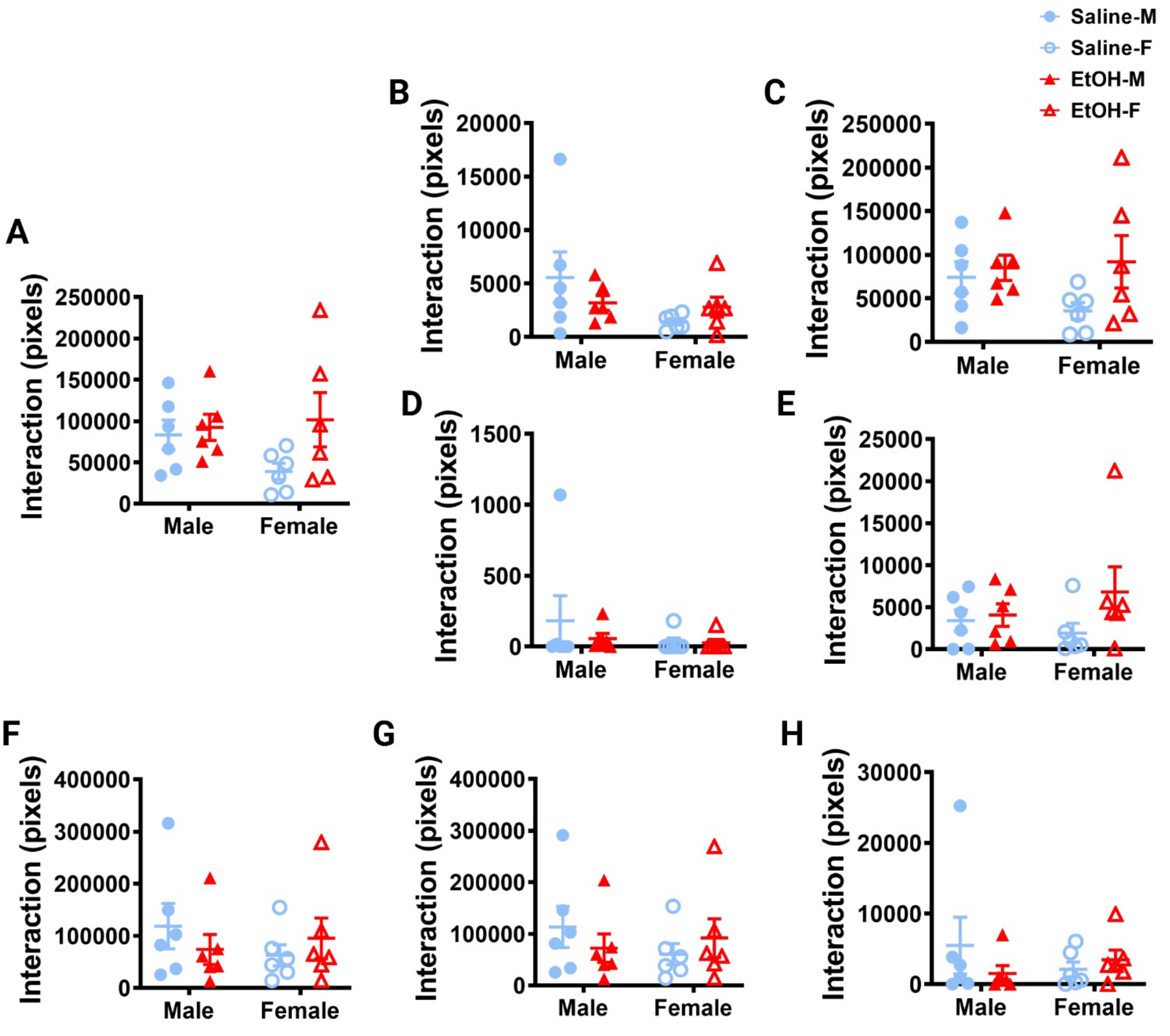

Supplementary Figure 2: ML and PCL Microglia-Purkinje Cell Interactions Without Normalization (A-H) Comparisons of microglia-Purkinje cell interactions when all timepoints are averaged together for microglia (MG) and Purkinje cells (PC) without normalization in the ML **(A-E)** and PCL **(F-H)** across conditions and sexes. In the ML, interactions for the whole MG (processes + somas) and whole PC (dendrites + branchpoints) **(A);** MG processes and PC branchpoints **(B);** MG processes and PC dendrites **(C);** MG somas and PC branchpoints **(D);** MG somas and PC dendrites **(E)** were unaffected. In the PCL, interactions for the whole MG (processes + somas) and PC somas **(F);** MG processes and PC somas **(G);** MG somas and PC somas **(H)** were unaffected. **A-H:** Two-way ANOVAs. Scale= 25μm
